# Circulating microRNAs as the Potential Diagnostic and Prognostic Biomarkers for Nasopharyngeal Carcinoma

**DOI:** 10.3390/genes13071160

**Published:** 2022-06-27

**Authors:** Thuy Ai Huyen Le, Thuan Duc Lao

**Affiliations:** Department of Pharmaceutical and Medical Biotechnology, Faculty of Biotechnology, Ho Chi Minh City Open University, Ho Chi Minh City 700000, Vietnam; thuy.lha@ou.edu.vn

**Keywords:** circulating microRNAs, extracellular miRNA, nasopharyngeal carcinoma, miRNA-based diagnosis

## Abstract

microRNAs are endogenous non-coding miRNAs, 19–25 nucleotides in length, that can be detected in the extracellular environment in stable forms, named circulating miRNAs (CIR-miRNAs). Since the first discovery of CIR-miRNAs, a large number of studies have demonstrated that the abnormal changes in its expression could be used to significantly distinguish nasopharyngeal carcinoma (NPC) from healthy cells. We herein reviewed and highlighted recent advances in the study of CIR-miRNAs in NPC, which pointed out the main components serving as promising and effective biomarkers for NPC diagnosis and prognosis. Furthermore, brief descriptions of its origin and unique characteristics are provided.

## 1. Introduction

Nasopharyngeal carcinoma (NPC), a malignant tumor that arises from the nasopharynx epithelium, is a highly curable disease if detected and diagnosed early. The absence of obvious symptoms in the early stages of NPC has made early diagnosis impossible; therefore, many patients are diagnosed at an advanced stage, leading to decreased survival [1,2]. That is why early diagnosis and screening play significant roles in increasing the success rate of NPC treatment as well as patient survival before it has progressed to an untreatable, ultimately fatal stage.

Biomarkers (biological markers) have gradually been recognized as measurable indicators in the diagnosis and prognosis of human diseases, including NPC [3]. To date, the main clinical practice, screening for NPC, is to detect the presence of Epstein–Barr virus (EBV), one of the etiological factors, via the detection of EBV DNA, EBV-VCA-IgA, EBNA1-IgA, and Rta-IgG [4,5,6]. The detection rate of NPC varies from 20–100%, leading to the varied efficacy of serology marker examination [7]. Oncoproteins, including EBV latent proteins (LMP-1, LMP-2A, and LMP-2B), and Epstein–Barr virus nuclear antigens (EBNA-1, -2, -3A, -3B, -3C, and –LP) encoded by EBV are also used to implicate the progression of nasopharyngeal tumorigenesis [8,9,10,11,12]. Notably, EBV is widespread in healthy people globally, infecting more than 90% of people [13,14]. The infection of EBV has also been reported in many different pathogeneses of human diseases, such as Burkitt’s lymphoma, Hodgkin’s disease, non-Hodgkin’s lymphoma, breast cancer, gastric cancer, and so on [6,12,15]. This is due mainly to the unsuitability for early diagnosis and screening of NPC based on EBV detection. Therefore, it is urgent that we identify novel and potentially reliable biomarkers that aim towards early clinical diagnosis as well as screening of NPC.

Apart from oncoproteins encoded by EBV, several proteins involved in the regulation of many tumorigenesis processes, including assisting tumor growth and survival, as well as tumor cell immigration and invasion, have been proven to be secreted into the tumor environment [11,16]. Many secreted proteins were reported to be substantially expressed in NPC cells compared to healthy ones, including heat shock protein 70 (Hsp70), plasminogen activator inhibitor 1 (PAI-1), fibronectin, MAC-2-binding protein (MAC-2 BP), and so on [11,16,17,18,19,20]. Despite the fact that several studies have identified these secreted proteins as possible biomarkers for NPC diagnosis and prognosis, there are still a number of issues that need to be addressed before these proteomic biomarkers may be used in clinical diagnosis and prognosis. Primarily, the concentrations of expressed proteins are dynamic, fluctuating dramatically at times with regard to stress, illness, and/or treatment. Secondly, quantitative discoveries of proteins require advanced high-throughput technologies, which, therefore, lead to many difficulties associated with sample isolation [11,21,22,23]. Finally, similar to oncoproteins encoded by EBV, there is a lack of further clinical investigations and validations.

Many efforts are now being undertaken to determine prospective biomarkers for the early diagnosis and screening of NPC that are based on the detection of microRNAs (miRNAs), etiological factors of tumorigenesis [2,24]. It is becoming increasingly evident that the aberrant expression of miRNAs, which function as oncogenic miRNAs, known as “oncomirs”, and tumor suppressor miRNAs, resulting in numerous cancer-associated phenomena, such as anti-apoptosis, proliferation, migration, metastasis, and so on, are responsible for numerous human pathogeneses, including nasopharyngeal tumorigenesis [12,14,24,25,26,27]. Thus far, many researchers have studied to identify the signature of miRNA expression in nasopharyngeal tumorigenesis [27,28,29,30]. The study of Zhu et al., which pointed out that miR-106a-5p is markedly up-regulated in NPC specimens, using methods of quantitative real-time PCR, miRNA microarray, and TCGA database analysis findings, is case in point [28]. In their report, they claimed that overexpression of miR-106a-5p was linked to advanced stages, recurrence, and poor clinical outcomes in NPC patients. Furthermore, through its target gene, *BTG3* (*BTG anti-proliferation factor 3*), and activation of autophagy-regulating MAPK signaling, its overexpression inhibits autophagy and promotes the malignant phenotype of nasopharyngeal cancer cells [28]. Their observations may lead to novel insights into the nasopharyngeal carcinoma pathogenesis. Recently, miRNAs existing in body fluids, such as plasma, serum, saliva, and so on, also known as circulating miRNA (CIR-miRNAs) or extracellular miRNA (EC-miRNA), have attracted interest in the investigation of biomarkers for NPC [14,27,31,32]. The discovery of CIR-miRNAs was unanticipated, thus, understanding their significant characteristics and clinical usefulness in cancer diagnosis and screening is urgent. In this article, we focus on summarizing the unique characteristics and usefulness of CIR-miRNAs in NPC diagnosis, screening, and therapy.

## 2. Brief Introduction of miRNAs and Their Regulation

**Figure 1:**
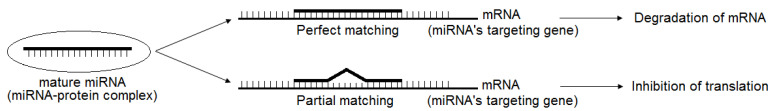
The regulation of gene expression through the binding of miRNA.

Since the first miRNA, namely lin-4, in *Caenorhabditis elegans*, was discovered by Victor Ambros and colleagues, the expression patterns of miRNAs have been reported as having fundamental roles in cancer initiation development and progression [14,24,25,26,27,28,30,33]. miRNAs are a family of small non-coding miRNAs, 19–25 nucleotides in length, which play multiple significant roles in a variety of biological processes, including cell proliferation, differentiation, metabolism, stress response, as well as apoptosis, via the regulation of its target genes [24,25,26,27]. The majority of post-transcriptional regulation of miRNA target genes is accomplished through the binding of miRNAs to their target sequences at the 3′-untranslated region (3′-UTR), eventually resulting in the degradation and/or repression of mRNA [24,25,26,34] (Figure 1). Additionally, miRNAs attach with other regions of target genes, including 5′-UTR, as well as promoter and coding regions [24,35]. According to Lewis et al. (2005), more than a third of human genes appear to have been selectively pressured to keep their miRNA seed pairing [36]. The expression of mRNA is down-regulated as a result of the pairing between miRNA and mRNA. This interaction is formed by the binding between a “seed” region located at the 5′ UTR of miRNAs and 3′ UTR of target mRNA conforming to the Watson–Crick rule [24,36]. Four types of sites, including 6 mer-site, 7 mer-A1 site, 7 mer-A8 site, and 8 mer-site, have been identified [24,36,37]. Due to its broad range of target genes, current research has provided much evidence proving that individual miRNA serves as the mediator of biological gene networks related to many biological functions by acting at the post-transcriptional level. According to the data of miRbase (https://www.mirbase.org/, accessed on 30 April 2022 there are more than 4.700 known human miRNAs (within the prefix hsa-) that have been identified.

The cellular biogenesis of miRNAs involves both the canonical pathway and non-canonical pathway [24,25,38]. Mature miRNA originates from primary miRNA (pri-miRNA) and precursor miRNA (pre-miRNA). The structure of 5′-7 methyl-guanosine capped and 3′ polyadenylated pri-miRNA is transcribed from the intragenic region or intronic region by RNA-polymerase II [24,39,40]. Pri-miRNA is subsequently cleaved into a ~60–70 nucleotide hair-spin structural pre-miRNA by Drosha and Dicer [41,42]. Pre-miRNA is exported into the cytoplasm for further processing by exportin-5 and Ras-related nuclear protein guanosine triphosphate (RAN-GTP) [43]. In the cytoplasm, the stem loop of pre-miRNA is removed by complex of Dicer/transactivation-responsive RNA-binding protein (TRBP) to form a 20–22 nucleotide-long miRNA duplex, which consists of a 5′ phosphorylated strand and 3′overhang, named the mature miRNA guide strand (miRNA) and complementary passenger strand (miRNA*). In the final step, the duplex of miRNA/miRNA* is loaded into an Argonaute protein (Ago protein) to generate an RNA-induced silencing complex (RISC), whereas the other strand (passenger strand) is degraded [24,25,44]. This mature miRNA cooperates with RISC to regulate the expression of target genes through the perfect binding or partial binding of the “seed” region of miRNA to the target mRNA’s 3′UTR according to the principle of Watson–Crick complementary base pairing, resulting in the degradation of mRNA and inhibition of translation [45].

## 3. Circulating miRNAs (CIR-miRNAs), Their Origin and Unique Characteristics

Tumor-associated miRNAs primarily detected in cellular environments can help to more accurately diagnose and monitor human cancer [34,46]. Recently, a handful of CIR-miRNAs, also known as EC-miRNA, have been detected outside of the cellular environment (extracellular environment), including in various bodily fluids [34,47,48]. CIR-miRNAs are not only detected in whole blood and plasma, but also in saliva, tears, urine, breast milk, follicular fluid, semen, and so on [34,47,48]. Since the discovery of CIR-miRNAs, these findings represent a novel approach for human cancer diagnosis and screening due to their being less invasive or non-invasive. For example, Wen et al. identified two miRNA signatures for the highly accurate diagnosis and differential diagnosis of patients with NPC, which represented novel serological biomarkers and potential therapeutic targets for NPC [27]. When studying the expression of serum miRNAs in 74 cases of patients with nasopharyngeal carcinoma and 57 cases of non-cancerous volunteers, they found that miR-17, miR-20a, miR-29c, and miR-223 among CIR-miRNAs were exclusively expressed in the sera of non-cancerous samples compared with that of NPC patients. Additionally, high sensitivity and specificity were recorded by calculating Ct differences, which have been shown to distinguish between NPC cases and controls. Based on their results, the four CIR-miRNAs, miR-17, miR-20a, miR-29c, and miR-223, may provide a novel strategy for NPC diagnosis and screening [14]. The expression patterns of CIR-miRNAs from different types of body fluids reflect the NPC status. Therefore, exploring novel strategies for NPC invasive markers from body fluids is promising, even though challenging, for early diagnosis and screening of NPC.

**Figure 2:**
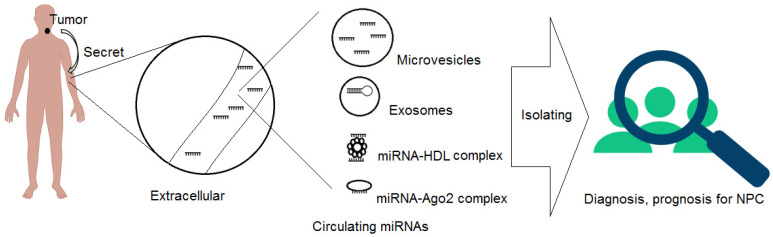
MicroRNAs are exported from cells into circulation by many mechanisms.

The mechanism by which CIR-miRNAs enters the extracellular environment is not fully understood. Until now, two major types of CIR-miRNAs, vesicle-associated and non-vesicle-associated forms, have been identified [49]. The non-vesicle-associated type includes CIR-miRNAs that are freely circulating, bound to specific proteins, and that are enclosed in the extracellular environment [50] (Figure 2).

Pre-miRNAs and mature miRNAs can be incorporated into small vesicles called exosomes (50–90 nm), which originate from endosomes and are then released from cells by fusing with the plasma membrane, or they can be released by microvesicles (~1 μm) that are released from the cell by blebbing of the plasma membrane [49,50,51]. Other major studies confirmed that CIR-miRNAs also exist in the non-vesicle-associated form (microparticle-free form). These miRNAs can be incorporated into high-density lipoproteins, or bound to RNA-binding proteins such as Argonaute2 (Ago2), which is the central protein of miRNA-mediated interference, and, together with GW182, was shown to be in charge of extracellular miRNA protection and transport [49,51,52]. It is unclear how these miRNA–protein complexes exit the cell. These miRNAs, exhibiting characteristics from tumor cells, can be produced in two ways: passively, as by-products of dying cells, or actively, in a miRNA-specific manner, via interactions with certain membrane channels or proteins [49,51].

CIR-miRNAs have several unique characteristics [53]. Unlike cellular miRNAs and mRNAs, which are degraded in the extracellular environment in a few seconds, CIR-miRNAs surprisingly persist for lengthy periods of time under adverse environments, such as extreme pH and RNAse digestion [34,54]. Mitchell et al. (2008) found that human plasma and serum contain miRNAs, which present in a remarkably stable form and are resistant to the activities of endogenous RNAse. They also investigated the stability of miRNAs in plasma by incubating plasma at room temperature for up to 24 h and subjecting it to up to eight cycles of freeze–thawing [54]. In the study of Turchinovich et al. (2011), the authors confirmed that circulating mature miRNA is extremely stable in blood plasma and cell culture media. Additionally, CIR-miRNAs can also be maintained in the extracellular environment for at least two months following cell lysis. miRNAs are thought to have originated as by-products of dying cells, and remain stable in the extracellular space for a long time due to the great stability of the Ago2/miRNA complex [55]. These findings suggest that these CIR-miRNAs use certain protective mechanisms to avoid being attacked by RNase in the extracellular environment. These circulating small molecules have unique properties that make them attractive candidates for use as biomarkers in a variety of liquid biopsies, such as saliva, cerebrospinal fluid, ascites, urine, breast milk, and sperm, for cancer diagnosis and prognosis [30,49]. However, mechanisms by which CIR-miRNAs exhibit exceptional stability in the RNase-rich environment of the blood and other bio-fluids are not well understood.

## 4. CIR-miRNAs as Diagnosis and Prognosis Biomarkers for NPC

Can the expression profile of CIR-miRNAs be identified as an effective biomarker for NPC diagnosis and prognosis? Recently, independent studies have successfully proven that CIR-miRNAs, extracted from whole blood and plasma, as well as serum, can be used as biomarkers for the diagnosis of NPC due to their stability and predictive properties. In the current revision, we focus on the results of recent studies that revealed the function of CIR-miRNAs as diagnosis and prognosis biomarkers for NPC. From the expression of many valuable CIR-miRNAs, including hsa-miR-let-7b-5p, hsa-miR-140-3p, hsa-miR-144-3p, hsa-miR-17-5p, hsa-miR-20a-5p, hsa-miR-20b-5p, hsa-miR-205-5p, hsa-miR-22, hsa-miR-miR-572, hsa-miR-638, hsa-miR-1234, and so on, extracted from whole blood, plasma, and serum, it has been reported that microRNAs possess great diagnostic power in NPC [14,27,56,57,58] (Table 1).

miRNA profiles extracted from whole blood in NPC patients were investigated by Wen et al. (2019). In their study, the training group-1, containing 84 NPC samples and 21 healthy samples, and the validation group-1, consisting of 36 NPC samples and 9 healthy samples, were established and enrolled in an NPC diagnostic model and Lasso regression to identify miRNA profiles to diagnose NPC. The profile of eight CIR-miRNAs, including hsa-miR-188-5p, hsa-miR-1908, hsa-miR-3196, hsa-miR-3935, hsa-miR-4284, hsa-miR-4433-5p, hsa-miR-4665-3p, and hsa-miR-513b, were identified as highly accurate in the diagnosis of patients with NPC from healthy ones. The same results of accurate diagnosis were also observed in validation group-1. Additionally, they also reported that high levels of hsa-miR-4790-3p, hsa-miR-188-5p, hsa-miR-5583-5p, and hsa-miR-3615 were frequently observed in NPC samples than in healthy samples. In their study, they compiled a panel of signatures for NPC diagnosis, and their results demonstrated an accuracy of 97.14%, sensitivity of 96.43%, specificity of 100%, positive predictive value of 100%, and negative predictive value of 87.5% [27].

Furthermore, tumor cells have been reported to release miRNAs into the circulation, serum, and plasma; therefore, they have been proposed as potential sources for CIR-miRNAs isolation [54]. Zhang et al. (2020) designed a study with four-stage validation, which consisted of the screening, training, testing, and external validation stages, to identify potential biomarkers for NPC diagnosis by quantitative reverse transcription polymerase chain reaction [56]. In their study, in the validation stage, seven plasma CIR-miRNAs, including hsa-let-7b-5p, hsa-miR-140-3p, hsa-miR-144-3p, hsa-miR-17-5p, hsa-miR-20a-5p, hsa-miR-20b-5p, and hsa-miR-205-5p, were identified to be significantly up-regulated in NPC patients compared with non-cancerous samples. Additionally, among these seven CIR-miRNAs, hsa-let-7b-5p was investigated to be significantly higher in the group of EBV-infected NPC samples, compared with non-EBV-infected NPC samples and healthy samples [56]. In the study of Liu et al. (2013), the combination of five plasma miRNAs, including hsa-miR-16, miR-21, hsa-miR-24, hsa-miR-155, and hsa-miR-378, resulted in values of sensitivity and specificity reaching 87.7% and 82.0%, respectively, for NPC diagnosis. In their study, they also reported that the specificity was reduced when removing hsa-miR-16 in their combination [59]. Collectively, combining panels of CIR-miRNAs can increase the sensitivity and specificity, and thus the accuracy, of CIR-miRNA biomarkers.

miRNA exist not only in blood fluid, such as whole blood, plasma, and serum, but are also detected in human saliva in a stable extracellular form [60]. Wu et al. (2019) was the first to investigate salivary miRNAs as potential indicators for nasopharyngeal cancer. In their study, they highlighted the potential salivary miRNAs as biomarkers for the detection of NPC based on the evaluation of miRNA expression in 22 saliva samples from NPC patients, and 25 healthy controls using microarray miRNA expression. They found that 12 miRNAs were significantly down-regulated in the saliva of NPC patients compared to healthy controls, with high accuracy. Additionally, the regulatory network for differentially expressed miRNAs was also successfully predicted. In their prediction, 11 out of 12 CIR-miRNAs, excepted for hsa-miR-4730, were incorporated into enriched GO pathways. Many target genes, such as platelet-derived growth factor receptor α (PDGFRA), Ras-related C3 botulinum toxin substrate 1 (RAC1), inhibitor of nuclear factor kappa B kinase subunit γ (IKBKG), X-linked inhibitor of apoptosis protein (XIAP), and protein phosphatase, Mg^2+/^Mn^2+^-dependent 1D (PPM1D), and so on, were simultaneously regulated by hsa-miR-937-5p, hsa-miR-650, hsa-miR-3612, hsa-miR-4478, hsa-miR-4259, hsa-miR-3714, hsa-miR-1203, hsa-miR-30b-3p, hsa-miR-1321, hsa-miR-1202, and hsa-miR-575 [31]. These findings suggest that differentially expressed saliva miRNAs may play a key role in NPC by targeting their target genes, which are linked to several important pathways.

It is emphasized that CIR-miRNAs profiles could help distinguish nasopharyngeal tumors from other head and neck tumors. In the study of Wen et al. (2019), they successfully identified and validated 16 miRNA signatures to differentiate NPC in comparison with other tumors located in the region of head and neck, and in non-cancerous samples. Additionally, this diagnostic model has an accuracy rate of 100%, specificity of 100%, and sensitivity of 100%. [27]. These results show that the profiles of 16 miRNAs could correctly distinguish NPC patients from head and neck tumor patients and heathy controls. Therefore, it is possible to apply CIR-miRNA non-invasive marker profiles to diagnose NPC.

As prognostic indicators, a stable and circulating presence in non-invasive specimens is also a characteristic aim for prognostic applications. Liu et al. (2014) conducted models to investigate the prognostic value of serum miRNAs in patients with NPC. They identified that four CIR-miRNAs, hsa-miR-22, hsa-miR-miR-572, hsa-miR-638, and hsa-miR-1234, added to the prognostic value to the stage system of TNM. In their results, the analysis of ROC indicated that the model combining the miRNA signature and TNM stage had improved the prognostic value for overall survival (area under ROC (AUROC): 0.69 vs. 0.60, *p* = 0.001; AUROC: 0.69 vs. 0.63, *p* = 0.008) and distant metastasis-free survival (AUROC: 0.71 vs. 0.60, *p* < 0.001; AUROC: 0.71 vs. 0.65, *p* = 0.005) relative to the TNM stage-alone model or miRNA signature-alone model in the training set, which may lead to more personalized therapy [56]. Finding a miRNA profile as an indicator of prognosis, might help to identify individuals who would benefit from more aggressive therapy and, as a result, enhance NPC patient survival. Collectively, these findings are encouraging in terms of using these CIR-miRNAs as a biomarker for the diagnosis and prognosis of NPC.

Referring to miRNA-derived EBV, which is encoded in the host cells, miRNA-derived EBV are transcribed from two regions of the EBV genome, BamH I fragment H rightward open reading frame 1(BHRF1)-cluster and BamH I fragment A rightward transcript (BART)-cluster 1, 2 [61,62]. The first cluster, BHRF1, which produces three miRNA precursors that subsequently encode four mature miRNAs, was discovered by Pfeffer et al. (2004). The second cluster, BART, which produces 22 precursors and encodes 40 mature miRNAs, was also found [60,61,62]. To date, many studies have revealed that EBV miRNAs play crucial roles in immune evasion, proliferation, apoptosis, invasion, and metastasis [61,63,64]. It is noted that EBV miRNAs, especially circulating EBV miRNA, could serve as biomarkers in NPC [61,62]. Recently, the clinical value of EBV-derived miRNAs for diagnosis and prognosis of EBV-positive nasopharyngeal tumors has been investigated. It has been reported that the plasma levels of BART7-3p and miR-BART13-3p are highly reflective of NPC diagnosis [65]. In their report, quantitative PCR was applied to evaluate the plasma levels of EBV DNA, miR-BART7-3p, and miR-BART13-3p, in 483 treatment-naïve NPC patients, compared to 243 controls. miR-BART7-3p and miR-BART13-3p were detected in 96.1% and 97.7% of NPC cases, whereas only 3.9% and 3.9% were detected in healthy controls, respectively. miR-BART13-3p values of sensitivity and specificity were 97.9% and 96.7%, respectively. The corresponding values for miR-BART7-3p were 96.1% and 96.7%. It is noted that the prognostic performance was assessed by comparing levels to distant metastatic rates during a 55-month follow-up of 245 NPC patients with radiotherapy treatment. After a four-year follow-up, the value of distant metastasis-free survival rates were 89.7% and 61.4% in subjects with detectable miR-BART7-3p and miR-BART13-3p, respectively. Additionally, at diagnosis and following radiation, a combination of plasma levels of miR-BART7-3p and EBV DNA could assist in stratifying individuals based on their risk of poor DMFS [65]. Other study, Wardana et al. (2020) found that circulating miR-BART-7 levels measured in peripheral blood samples can be used as a promising predictor for the clinical outcome and prognosis in NPC patients [66]. Based on these findings, circulating EBV miR-BART7 and miR-BART13 will shed light on the potential serological biomarkers for diagnosis and prognosis of EBV-positive nasopharyngeal malignancies. From their panel of EBV miRNAs, Gao et al. reported that plasma levels of BART2-3P, BART2-5P, BART5-3P, BART5-5P, BART6-3P, BART8-3P, BART9-5P, BART17-5P, BART19-3P, and BART20-3P were significantly increased. Additionally, EBV miRNAs, such as BART8-3P and BART10-3P, could potentially be employed as complementary serological markers if EBV DNA is beyond the lower detection limit or undetectable in plasma samples [67]. Most importantly, EBV miRNA research is still in its early stages, with only a few studies confirming that EBV miRNAs are abundantly expressed in EBV-associated tumors. Better understanding and evaluating, therefore, of the levels of EBV miRNAs might establish novel biomarkers for NPC diagnosis and prognosis. Furthermore, there is a great need for more research aiming to determine the diagnostic and prognostic value of EBV miRNAs in follow-up management of nasopharyngeal patients.

## 5. Challenges of Circulating miRNAs as Indicators for Diagnosis and Prognosis

Though there are many advantages of using CIR-miRNAs, such as their stability, particular expression profiles in different stages of cancers, and so on, there are still challenges to overcome before clinical application. First, because of their low concentration in the extracellular environment, detecting and evaluating CIR-miRNAs is certainly difficult [49]. Other RNAs in plasma or serum also exist; therefore, from analysis results, it is difficult to distinguish CIR-miRNAs from other RNAs. Second, many methods of detecting and measuring levels of CIR-miRNAs, including qRT-PCR, miRNA microarray, and next-generation sequencing (NGS) have been applied [14,27,31,55,56,57,58]. Each method might produce different findings; thus, unifying methodologies and eliminating variance is critical. An additional obstacle is that the understanding of CIR-miRNAs functions, as well as the relationship among CIR-miRNAs and the Epstein–Barr virus (EBV), is still limited. To date, a standard tool for NPC screening has been based on the detection of IgA antibodies against EBV capsid antigen (VCA/IgA) and early antigen (EA/IgA) [27,68]. However, the specificity and sensitivity of combining VCA/IgA and EA/IgA are 50.9% and 95.2%, respectively. Its low sensitivity reflects that EBV infection is involved in other hematological and epithelial malignancies, such as Hodgkin’s lymphoma, Burkitt’s lymphoma, and other tumors that originate from the oral epithelium, oropharynx, larynx, and so on [69,70]. Therefore, differentiating the CIR-miRNAs profiles between NPC and EBV-related human tumors, including head and neck tumors and esophagus carcinomas, remains a major challenge. Thus, more multicenter prospective trials to verify these diagnostic markers for NPC are still required.

## 6. Conclusions and Perspectives

The existence of CIR-miRNAs, extracted from human bodily fluid, including whole blood, plasma, serum, salivary, and so on, exhibits the promising and valuable potential of serving as diagnostic and prognostic biomarkers for human cancer, including NPC. CIR-miRNAs are proven to persist for a long time under harsh environmental conditions, meaning they could be effectively applied in clinical environments. However, many obstacles need to be solved urgently. The development of CIR-miRNAs-based diagnostic and prognostic tools for NPC is a lengthy process, hence larger studies, which promote sensitivity and specificity, are necessary.

## Figures and Tables

**Table 1:** The abnormal expression of CIR-miRNAs in NPC.

Year	CIR-miRNAs candidate	Description	Source	Reference
2020	hsa-miR-let-7b-5p, hsa-miR-140-3p, hsa-miR-144-3p, hsa-miR-17-5p, hsa-miR-20a-5p, hsa-miR-20b-5p, hsa-miR-205-5p	The panel of 7 miRNA, extracted from plasma, performed better in distinguishing NPC patients from healthy controls, the sensitivity and specificity being 0.74 and 0.76, respectively.	Plasma	[56]
2019	hsa-miR-188-5p, hsa-miR-1908, hsa-miR-3196, hsa-miR-3935, hsa-miR-4284, hsa-miR-4433-5p, hsa-miR-4665-3p, hsa-miR-513b	Theses 8 miRNA signatures diagnosed NPC with an accuracy of 97.14%, sensitivity of 96.43%, specificity of 100%, positive predictive value of 100%, and negative predictive value of 87.5% in a group of 84 NPC samples and 21 healthy samples.	Whole blood	[27]
2019	hsa-miR-937-5p, hsa-miR-650, hsa-miR-3612, hsa-miR-4478, hsa-miR-4259, hsa-miR-3714, hsa-miR-4730, hsa-miR-1203, hsa-miR-30b-3p, hsa-miR-1321, hsa-miR-1202, hsa-miR-575	These CIR-miRNAs were significantly down-regulated in saliva of NPC patients compared to healthy controls, detected by miRNA microarray platform with the high accuracy (sensitivity = 100.00%, specificity = 96.00%).	Salivary	[31]
2014	hsa-miR-22, hsa-miR-miR-572, hsa-miR-638, hsa-miR-1234	Different changes were observed in the serum of patients with NPC. The value of prognosis of the TNM staging system was reported. The patients of with high-risk scores had poorer overall survival and low metastasis-free survival than those with the patients with low-risk scores.	Serum	[57]
2014	hsa-miR-548q, hsa-miR-483-5p	miR-548q and miR-483-5p highly expressed in NPC cell lines and 31 plasma samples from NPC patients, compared with 19 non-cancerous controls. Combining these 2 CIR-miRNAs resulted in 67.1% sensitivity and 68.0% specificity.	Plasma	[58]
2014	hsa-miR-483-5p, hsa-miR-103, hsa-miR-29a	Differentially expressed CIR-miRNAs were identified as being effective biomarkers for predicting survival in NPC patients.	Plasma	[26]
2013	hsa-miR-16, miR-21, hsa-miR-24, hsa-miR-155, hsa-and miR-378	Sensitivity and specificity reached 87.7% and 82.0%, respectively, when combining the panel of CIR-miRNAs, hsa-miR-16, miR-21, hsa-miR-24, hsa-miR-155, hsa-and miR-378.	Plasma	[59]
2012	hsa-miR-17, hsa-miR-20a, hsa-miR-29c, hsa-miR-223	miRNAs were differentially expressed in the serum of 20 NPC patients compared with that of 20 non-cancerous controls. Using these 4 CIR-miRNAs, a diagnostic value with sensitivity of 97.3% and specificity of 96.5% was established.	Serum	[14]

## Data Availability

Not applicable.
